# Evidence for adaptive introgression of exons across a hybrid swarm in deer

**DOI:** 10.1186/s12862-019-1497-x

**Published:** 2019-11-04

**Authors:** Margaret L. Haines, Gordon Luikart, Stephen J. Amish, Seth Smith, Emily K. Latch

**Affiliations:** 1 Behavioral and Molecular Ecology Research Group, Department of Biological Sciences, University of Wisconsin-Milwaukee, Milwaukee, WI 53211 USA; 2 Montana Conservation Genomics Laboratory, Division of Biological Sciences, The University of Montana, 32 Campus Drive, Missoula, MT 59812 USA; 3 Montana Conservation Genomics Laboratory, Flathead Lake Biological Station, Division of Biological Sciences, The University of Montana, 32125 Bio Station Lane, Polson, MT 59860 USA

**Keywords:** Cascade Range, Hybridization, *Odocoileus*, Secondary contact, Admixture, SNPs

## Abstract

**Background:**

Secondary contact between closely related lineages can result in a variety of outcomes, including hybridization, depending upon the strength of reproductive barriers. By examining the extent to which different parts of the genome introgress, it is possible to infer the strength of selection and gain insight into the evolutionary trajectory of lineages. Following secondary contact approximately 8000 years ago in the Pacific Northwest, mule deer (*Odocoileus hemionus hemionus*) and black-tailed deer (*O. h. columbianus*) formed a hybrid swarm along the Cascade mountain range despite substantial differences in body size (up to two times) and habitat preference. In this study, we examined genetic population structure, extent of introgression, and selection pressures in freely interbreeding populations of mule deer and black-tailed deer using mitochondrial DNA sequences, 9 microsatellite loci, and 95 SNPs from protein-coding genes.

**Results:**

We observed bi-directional hybridization and classified approximately one third of the 172 individuals as hybrids, almost all of which were beyond the F1 generation. High genetic differentiation between black-tailed deer and mule deer at protein-coding genes suggests that there is positive divergent selection, though selection on these loci is relatively weak. Contrary to predictions, there was not greater selection on protein-coding genes thought to be associated with immune function and mate choice. Geographic cline analyses were consistent across genetic markers, suggesting long-term stability (over hundreds of generations), and indicated that the center of the hybrid swarm is 20-30 km to the east of the Cascades ridgeline, where there is a steep ecological transition from wet, forested habitat to dry, scrub habitat.

**Conclusions:**

Our data are consistent with a genetic boundary between mule deer and black-tailed deer that is porous but maintained by many loci under weak selection having a substantial cumulative effect. The absence of clear reproductive barriers and the consistent centering of geographic clines at a sharp ecotone suggests that ecology is a driver of hybrid swarm dynamics. Adaptive introgression in this study (and others) promotes gene flow and provides valuable insight into selection strength on specific genes and the evolutionary trajectory of hybridizing taxa.

**Electronic supplementary material:**

The online version of this article (10.1186/s12862-019-1497-x) contains supplementary material, which is available to authorized users.

## Background

Hybrid zones, once thought to be “exceptional or absent in animals” [1], have been increasingly reported in wide array of fauna, with an average of 10% of animal species estimated to hybridize [2]. Hybrid zones often arise between recently diverged lineages [2–4] as a result of natural secondary contact caused by post-glacial range expansion or environmental disturbance [5, 6]. Hybrid zones can take many forms, largely depending upon the strength of reproductive barriers and their effect on hybrid fitness. If hybrids are less fit than parental lineages, reproductive barriers, particularly those associated with pre-mating isolation, will often be reinforced in order to minimize wasted mating efforts [4, 7–9]. Tension zones are an exception, where selection pressures against hybridization are offset by migration into the hybrid zone [10].

Hybrid speciation can occur when hybrids are more fit than parental lineages and hybrids preferentially mate with each other [11], though examples are rare (but see [12–14]). When hybrids display similar fitness to one or both parents, hybrids may freely interbreed with other hybrids and parental populations, causing a hybrid swarm to form [15]. We define a hybrid swarm as a population consisting of a mixture of parental types, F1 hybrids, and backcrosses (as defined by Grant [16], Arnold [17] and others). While hybrid swarms can lead to lineage collapse [18, 19], they can also remain stable [20–23]. These different outcomes of hybridization are not necessarily mutually exclusive but may represent different evolutionary stages [15].

The dynamics of hybrid zones are generally fluid, changing in response to selection pressures. Selection can act evenly across traits or differ for particular phenotypes, resulting in variable rates of gene introgression. Alleles that reduce viability or fertility in hybrids or contribute to assortative mating are expected to be under stronger selective pressures and therefore show limited introgression. Conversely, alleles that confer higher fitness in hybrids should spread quickly [24–27]. The rate of introgression of neutral alleles is complex; however, it should generally exceed that of alleles associated with reproductive barriers [10, 24, 28]. In stable hybrid zones, strong (negative) selection against introgression on parts of the genome are enough to prevent complete panmixia but too weak to prevent the formation of complete reproductive isolation (e.g. [29–33]).

Identifying which regions of the genome are under selection can help explain overall patterns of introgression and provide insight into the structure of hybrid zone. By quantifying selection strength on individual regions or genes, we can assess to what extent they contribute to reproductive isolation. Strong selection on even a small portion of the genome can have a large impact on hybrid zone dynamics [24, 34, 35]. For example, hybrid inviability is known to be caused by as few as two linked loci in monkeyflower [36] and a single locus in *Drosophila* [37]. Genes under selection and those physically linked to them show reduced levels of gene flow. When these genes are overrepresented in a particular part of the genome, they are known as genomic islands of divergence [28, 38]. When genomic islands expand via accumulation of hitchhiker loci, gene flow can become further restricted and parental populations continue to diverge [39–41]. High rates of gene flow can reverse the divergence process by weakening population structure. This is more common in early stages of divergence when selection is weak and limited to relatively few loci [42].

Not only can selection pressures differ across the genome, they can also vary over the landscape. In hybrid zones, positive selection on ecological adaptations in only part of the landscape can cause the hybrid zone to move in the direction of overall greater selection until selection is counter-balanced by selection for the opposite trait or a barrier to gene flow is reached [10, 43]. For example, in a study on Australian grasshoppers, genetic clines were shown to shift across a deforested landscape towards an area of regenerated forest, a known barrier to gene flow [44]. Once opposing selection pressures are at equilibrium, genetic clines can become co-localized, stabilizing the hybrid zone. By examining the position of genetic clines for multiple marker types with different mutation rates, it is possible to evaluate hybrid zone stability.

In this paper, we investigate the dynamics of a seemingly stable hybrid swarm between black-tailed deer *Odocoileus hemionus columbianus* (BTD) and mule deer *O. h. hemionus* (MD; [45]). These subspecies experienced long periods of allopatry during Pleistocene glaciations, with black-tailed deer retreating to coastal refugia along the northwest coast of the United States and mule deer shifting their distribution south [46]. Following the last glacial maximum (LGM) 18,000 years ago, both lineages expanded their ranges and came into secondary contact approximately 8000 years ago along the Cascade Mountains, located in the northwestern United States. These subspecies not only differ greatly in size (MD males can be more than two times larger than BTD males) and preferred habitat [47–49] but also display 6–7.7% genetic divergence at mitochondrial loci [46, 50, 51], which is greater than the levels of divergence commonly observed between sister species in mammals [52, 53]. A preference for intra-lineage mating has been predicted to maintain this deep genetic divergence between subspecies [54].

Although both BTD and MD bucks are highly mobile, with the ability to travel over 25 km to seek out conspecific mates [55, 56], hybridization continues to occur. Previous work using mitochondrial DNA (mtDNA) and neutral microsatellite loci has shown widespread, bi-directional introgression between BTD and MD, indicative of hybrid swarm formation [45]. However, patterns of gene flow in other loci, such as protein-coding genes, remain unexplored. Investigating introgression in protein-coding genes that are potentially under selection would provide critical insight into the mechanisms preventing lineage fusion and the future trajectory of the hybrid swarm.

This study explores the dynamics of the BTD-MD hybrid zone and the role of selection in maintaining its stability. First, we compared patterns of population genetic structure and introgression inferred from different molecular data types reflecting a range of evolutionary history – single nucleotide polymorphisms (SNPs) from protein-coding regions, microsatellite loci, and mtDNA sequences – to identify signatures of hybrid zone stability and predict the future trajectory of the hybrid swarm. Second, we quantified the strength of selection on protein-coding divergence. We predicted that genes involved with disease resistance and mate discrimination in ungulates (e.g. olfaction) would be important for maintaining species boundaries ([57] and references therein) and therefore be under stronger selection than genes involved in general cell processes. Correspondingly, we predicted weak or no selection on genes associated with general cell processes, and expected SNPs within these genes to flow relatively freely across the hybrid zone. To test this hypothesis, we tested for selection on SNPs within protein-coding genes that exhibited high differentiation between BTD and MD. We compared ontogeny of SNPs potentially under selection to explore the role of potential candidate genes in maintaining species boundaries.

## Results

### Genetic structure

Both Bayesian and Maximum Likelihood analyses of the mitochondrial control region produced concordant topologies, dividing individuals into two well-supported clades, corresponding to BTD and MD (Additional file 1: Figure S1). Black-tailed deer were primarily found west of the Cascades and MD were east of the Cascades (Additional file 2: Figure S2). However, eight individuals west of the Cascades had MD mtDNA and 13 individuals east of Cascades had BTD mtDNA. The average mitochondrial genetic divergence between lineages was 6.4%, comparable to values observed in previous studies [46, 50, 51]. The BTD and MD mtDNA clades were comprised primarily of individuals sampled west and east of the Cascades, respectively. Within clades, there was weak substructure of haplotypes. The two white-tailed deer *O. virginianus* sequences collected from individuals outside the hybrid zone in eastern North America were embedded within MD. This was expected based on several previous studies that have showed low mitochondrial divergence between MD and white-tailed deer [58] and, in some instances, shared haplotypes [50, 59, 60].

### Hybrids and admixture

**Fig. 1:**
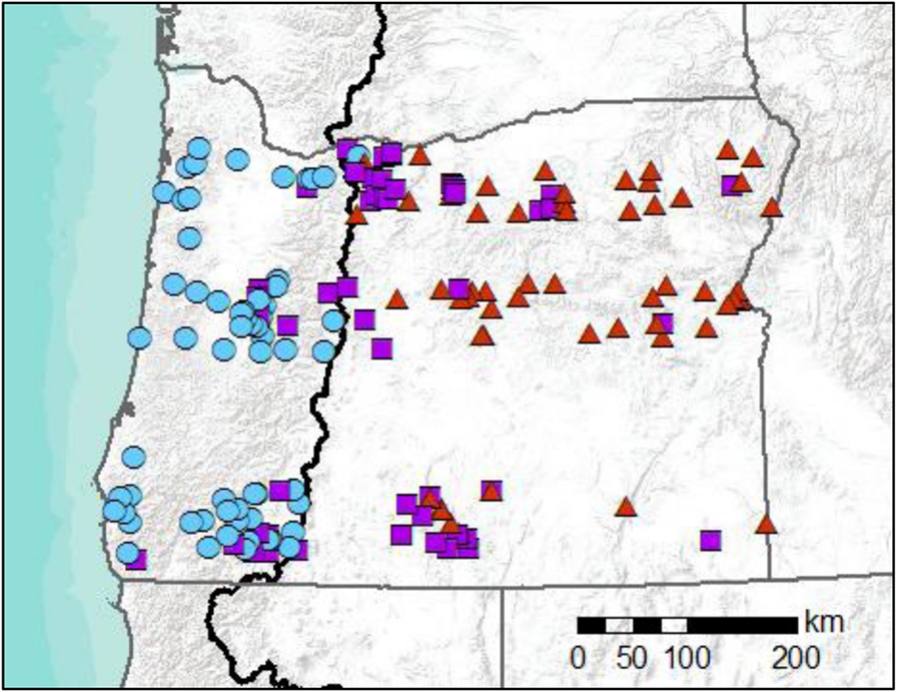
Collection localities for all *Odocoileus* individuals. Individuals are classified as black-tailed deer (blue circles), hybrids (purple squares), or mule deer (red triangles) based on fastStructure analysis of SNP data. The Cascades ridgeline is indicated by the bold black line. Map source: Esri

**Fig. 2:**
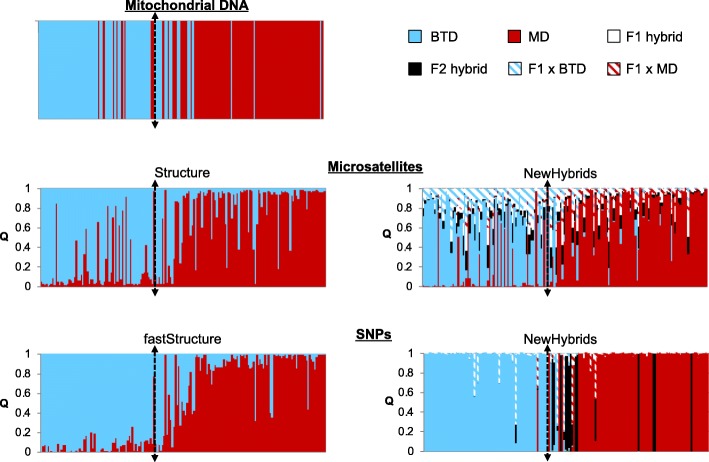
Individual assignments to black-tailed deer and mule deer lineages for mitochondrial DNA, microsatellite loci, and SNP loci. Samples are oriented west to east and the dashed black line indicates the location of the Cascade ridgeline. Individuals are represented by a single vertical line with the percentage of each color representing the individual proportion of membership (Q) for each lineage: black-tailed deer (blue) and mule deer (red). The NewHybrids plots have three additional categories: F1 hybrid (white), F2 hybrid (black), F1 x black-tailed deer (white and blue stripes), F1 x mule deer (white and red stripes)

Admixture analysis of the microsatellite and SNP data also showed strong support for two clusters corresponding to BTD and MD, with individuals consistently more clearly delineated using SNPs (Fig. 2). There was no substructure within clusters. Cut-offs for pure BTD and pure MD were calculated using simulated data and varied slightly between microsatellites and SNPs. For the STRUCTURE (microsatellites) and fastSTRUCTURE (SNPs) analyses, individuals with Q > 0.941 (microsatellites) or Q > 0.865 (SNPs) for the BTD cluster were classified as pure BTD and individuals with Q > 0.928 (microsatellites) or Q > 0.899 (SNPs) for the MD cluster were classified as pure MD; all other individuals were considered hybrids (Fig. 1, Additional file 2: Figure S2). Assignments were consistent among runs (standard errors in the range of 10^− 4^). Individuals assigned to a parental lineage typically belong to that mtDNA lineage while hybrids had both BTD and MD mtDNA. We did observe some evidence of mitochondrial capture. Three individuals assigned as pure BTD using both microsatellites and SNPs had MD mtDNA and two pure MD had BTD mtDNA. Assignments based on microsatellite and SNP datasets were the same for 100 of 172 individuals (36 BTD, 39 MD, and 25 hybrids). All mismatches occurred when an individual was classified as a BTD or MD for either microsatellites or SNPs and a hybrid in the other genetic dataset. Though there was some disparity between datasets, a paired t-test showed that the Q values for the microsatellite and SNP analyses were not significantly different (*p* = 0.09).

NewHybrids analyses were generally concordant with the STRUCTURE and fastSTRUCTURE analysis when the Uniform prior was applied (Fig. 2). There was little evidence of F1 individuals but high support for the presence of F2 individuals and some backcrossing. Hybrids from all categories (F1, F2, backcrosses) were scattered on both sides of the Cascades but were more concentrated closer to the ridgeline. SNP results were relatively unaffected by the choice of prior. In contrast, when we used a Jeffrey’s-like prior for the microsatellite loci, no individuals were assigned as pure MD (Q > 0.939 for MD) and 34 individuals were assigned as pure BTD (Q > 0.837) whereas the results using the uniform prior indicated that 30 individuals were pure MD (Q > 0.957) and only 15 individuals were pure BTD (Q > 0.876).

### Genetic diversity

Estimates of F_ST_ and D_est_ did not differ significantly between any pair of transects, permitting transects to be combined in subsequent analyses. There were more private alleles for pure BTD than pure MD or hybrids (non-overlapping 95% CIs) in the microsatellite dataset but the total number of alleles was comparable across groups (Table 1). Observed heterozygosity (H_O_) and expected heterozygosity (H_E_) for microsatellites were similar for pure BTD, pure MD and hybrids. For SNPs, H_E_ was significantly higher than H_O_ for MD based on 95% CIs. Microsatellite analysis showed a significant deficiency of heterozygotes in all groups (F_IS_ (BTD) = 0.126, 95% CIs 0.037–0.202; F_IS_ (MD) = 0.084, 95% CIs 0.018–0.173; F_IS_ (hybrids) = 0.109, 95% CIs 0.069–0.155; *p* < 0.01 for all groups). For SNPs, only F_IS_ for hybrids had a confidence interval that did not include 0 (F_IS_ = 0.219, 95% CIs 0.177–0.263, *p* < 0.01). Heterozygote deficiencies are likely due to nonrandom mating; we found no evidence for null alleles in this study or in other studies using these markers [45, 61], and it should not reflect ascertainment bias as roughly the same number of MD and BTD individuals were analyzed. Though positive F_IS_ values can reflect cryptic substructure, there was little support for substructure in the mtDNA data.

**Table 1:** Molecular genetic diversity of three Oregon deer lineages for 583 bp of the mitochondrial control region, nine microsatellites and 95 SNPs

		Black-tailed deer	Mule deer	Hybrids
mtDNA	N	79	93	-
	π	0.012 ± 0.00004	0.027 ± 0.00006	-
	H	0.96 ± 0.002	0.97 ± 0.001	-
Microsatellites	N	56	65	51
	A_R_	8.49 ± 1.46	6.76 ± 1.15	7.50 ± 1.37
	A_PR_	1.71 ± 0.53	0.33 ± 0.23	0.53 ± 0.25
	H_E_	0.70 ± 0.04	0.68 ± 0.05	0.69 ± 0.05
	H_O_	0.60 ± 0.05	0.64 ± 0.05	0.62 ± 0.05
SNPs	N	61	57	54
	H_E_	0.32 ± 0.02	0.26 ± 0.02	0.37 ± 0.01
	H_O_	0.32 ± 0.02	0.25 ± 0.02	0.29 ± 0.01

*N* Number of samples, *π* Nucleotide diversity, *H* Haplotype diversity, *A*_*R*_ Allelic richness, *A*_*PR*_ Private allelic richness, *H*_*E*_ Expected heterozygosity and *H*_*O*_ Observed heterozygosity for non-hybridized individuals. All values are ± SE

**Fig. 3:**
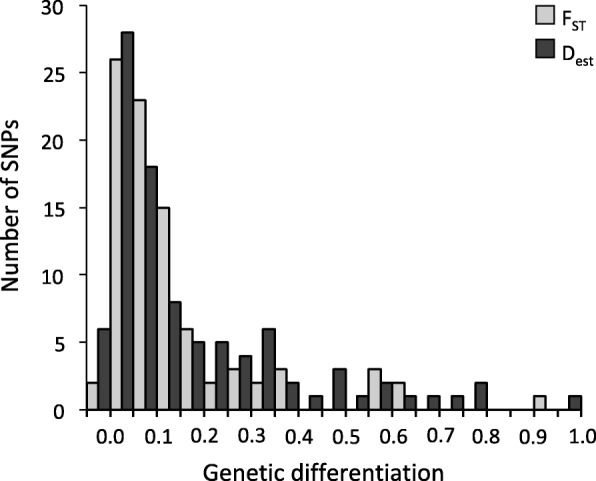
Distribution of estimates of genetic differentiation between ‘pure’ BTD and MD for 95 SNP loci. The number of SNPs was plotted against F_ST_ (light gray) and Dest (dark gray). Average genetic differentiation was 0.183 (F_ST_) and 0.180 (Dest)

Pure parental populations exhibited high genetic differentiation. Estimates of F_ST_ were significantly lower for microsatellites (0.070, 95% CI 0.040–0.107) than SNPs (0.182, 95% CI 0.142–0.228, Fig. 3). F_ST_ estimates for highly variable microsatellites are expected to be lower than estimates for SNPs, because their high heterozygosity keeps them far from fixation [62, 63]. D_est_, which is independent of within-population diversity [62, 64], was not significantly different between microsatellites (0.284, 95% CI 0.118–0.492) and SNPs (0.178, 95% CI 0.134–0.227). This indicates that populations share roughly the same proportion of allelic diversity and suggests that the level of divergence between parental lineages has remained relatively constant despite ongoing hybridization.

### Signatures of SNP selection

Contrary to our predictions, both methods of outlier detection (BayeScan and pcadapt) only identified a single SNP likely to be under selection. This SNP represents a non-synonymous mutation and was found within the gene EIF4G3, a likely component of the protein complex EIF4F which is involved in the recognition of the mRNA cap, ATP-dependent unwinding of the 5′ terminal secondary structure and recruitment of mRNA to the ribosome [65, 66]. This gene has elevated expression in testis in humans [67], rats [68], and mice [69], and EIF4G3 mutations can cause male infertility in mice [70]. Individuals identified as having pure BTD ancestry by the fastSTRUCTURE analysis were almost exclusively homozygous for the major allele and pure MD were predominantly homozygous for the minor allele. Hybrids displayed all combinations of alleles.

We also tested for selection by calculating pairwise F_ST_ and D_est_ between pure parental lineages for each locus (Fig. 3). The strength of selection was presumed to be positively correlated with the magnitude of genetic differentiation. The outlier locus EIF4G3 identified above had by far the highest differentiation for both metrics (F_ST_ = 0.930, D_est_ = 0.962). Regardless of whether SNPs were ranked by F_ST_ or D_est_, the top 10 % of SNPs included the same set of nine loci (Table 2). We classified each outlier SNP as a synonymous or non-synonymous mutation using ENSEMBL gene predictions in the BLAT tool [71] in the University of California Santa Cruz Genome Browser (https://genome.ucsc.edu/cgi-bin/hgGateway). Assuming linkage groups in *Odocoileus* are similar to those of *Bos taurus*, these outlier loci were scattered across five of the 29 chromosomes sampled. It is unlikely that any of these nine SNPs are linked because they are located at least 10 megabases apart (> 0.001 likelihood of linkage; [72]), and, in some instances, loci with no evidence of selection are found between them. Additionally, we found no evidence that selection was stronger on genes putatively involved with immune function and mate choice than those involved in general cell processes.

**Table 2:** Description and primary function of SNPs with highest estimates of F_ST_ (> 0.44) and D_est_ (> 0.52) and/or excess ancestry based on *bgc* analyses

Gene	F_ST_/D_est_	bgc	Chromosome^a^	Description	Putative function^b^	Synonymous mutation?
ANG2		x	10	Angiogenin 2	Nuclease activity	No
AP3B1	x	x	10	Adapter-related protein complex 3 beta 1	Cellular biogenesis; immune system	No
EIF4G3	x	x	2	Eukaryotic translation initiation factor 4 gamma 3	Transport	No
F9	x		X	Coagulation factor IX	Immune system	No
FUT8	x	x	10	Fucosyltransferase 8	Immune system	No
NLN	x		20	Neurolysin	Cell signalling	No
PLIN2	x		8	Perilipin-2	Metabolism	No
ROPN1L	x		20	Rhophilin associated tail protein 1 like	Reproduction	Yes
SCRG1	x		8	Scrapie-responsive protein 1	Immune system	No
TGFB3	x	x	10	Transforming growth factor beta 3	Cell development	No
TRPM3		x	8	Transient receptor potential cation channel subfamily M	Ion channel activity	Yes

^a^Chromosome based on the *Bos taurus* reference genome

^b^Gene function was based on NCBI gene report (http://www.ncbi.nlm.nih.gov)

### Geographic cline analyses

**Fig. 4:**
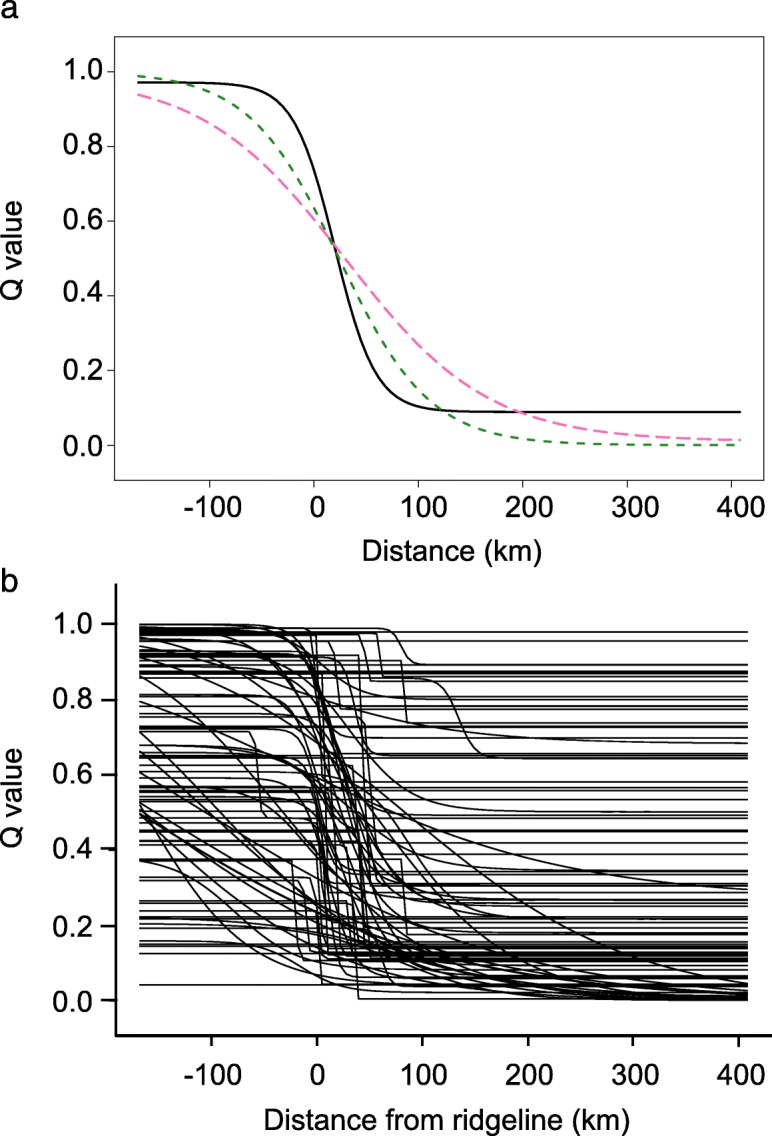
Geographic clines showing the transition from black-tailed deer (top left) to mule deer (bottom right) across the Cascade ranges for **a** mtDNA (solid black line), microsatellites (dashed pink line), and SNPs (dotted green line) and **b** the 74 SNPs showing a change in allele frequency between parental lineages. Microsatellite and SNP composition were inferred from Q values for the black-tailed deer cluster in STRUCTURE and fastSTRUCTURE, respectively

We fit geographic cline models to the mtDNA, microsatellite and SNP datasets in order to characterize cline shape. Based on AICc, models with fixed maximum and minimum values of 1 and 0 were selected for all datasets and cline tails were estimated for the SNP dataset only. Models predicted that the cline center for all datasets was significantly to the east of the Cascade ridgeline, with average cline center varying between + 20 and + 30 km (Fig. 4a). All individuals classified as pure BTD or pure MD across all three markers were found on the expected side of the cline center. The SNP dataset had the narrowest cline width (77 km) and was significantly narrower than the microsatellite cline (274 km) but not the mtDNA cline (174 km).

Within the SNP dataset, we could not reject the null model (i.e. no change in allele frequency across the landscape) for 21 SNPs. For the remaining 74 SNPs, the change in allele frequency from west to east occurred over a relatively narrow range, with an average slope of 0.10 ΔP/km (Fig. 4b). We observed the steepest slope for the SNP in the amino acid biosynthesis gene PSAT1 (2.7 ΔP/km; [73]), which was over three times steeper than the slope for any other SNP. Steep slopes represent a rapid change in allele frequency and suggest relatively strong selection. Cline slopes were not correlated with genetic differentiation between parental lineages (F_ST_: *r*^2^ = 0.0148; D_est_: *r*^2^ = 0.0005).

### Genomic cline analyses

**Fig. 5:**
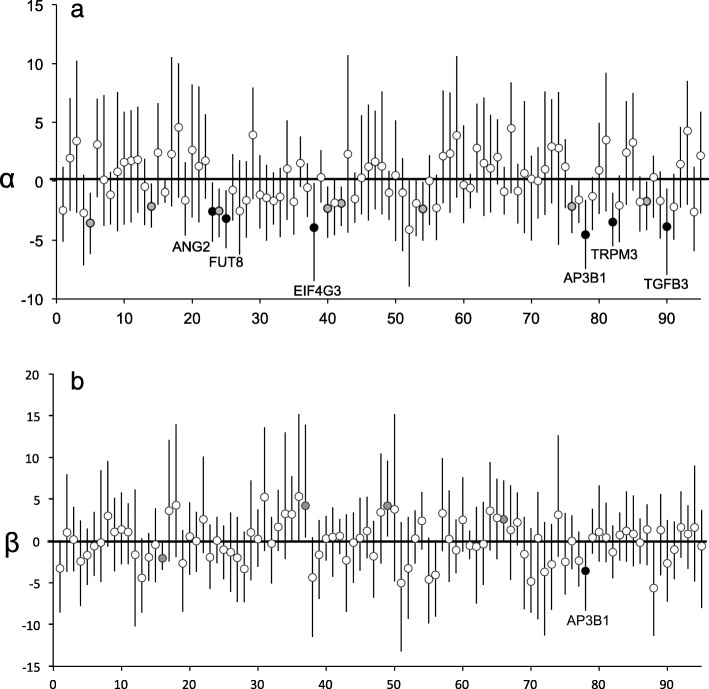
Potential candidate loci based on *bgc* genomic cline analyses. **a** Estimates of genomic cline center (α) with 95% credibility intervals (CI). Values below zero indicate greater black-tailed deer ancestry and values above zero indicate greater mule deer ancestry. Black dots indicate six loci with significant excess ancestry (95% CI does not include zero) and the difference in allele frequency between putative parental black-tailed deer and mule deer is > 0.5 (with gene names listed), grey dots indicate loci with significant excess ancestry and the difference in allele frequency between putative parental black-tailed deer and mule deer is < 0.5, and white dots indicate loci with no evidence of excess ancestry. **b** Estimates of genomic cline slope (β) with 95% credibility intervals. Values below zero indicate shallower slopes than expected and values above zero indicate steeper slopes. Black dots indicate loci with significantly shallower clines (95% CI does not include zero) and the difference in allele frequency between putative parental black-tailed deer and mule deer is > 0.5, grey dots indicate loci with significantly shallower clines and the difference in allele frequency between putative parental lineages is < 0.5, and white dots indicate loci with no evidence of slopes that deviate from expectations

Bayesian genomic cline analyses identified candidate genes that may influence the strength of reproductive barriers and/or increase local adaptation. Analyses on individual SNPs suggest excess ancestry from one parental population (α) for only a few SNPs and only a single locus with significant rates of change in allele frequency (β) across the hybrid zone. Using 95% confidence intervals, 14 loci generally had weak evidence of excess BTD ancestry, with mean values for α between − 5 and 0 (Fig. 5a). However, when loci with a small difference (< 0.5) between parental allele frequencies were excluded following Trier et al. [74], the number of loci with excess BTD ancestry decreased to six loci. Four of these loci were also identified as candidate loci based on F_ST_ (Table 2) and all six had F_ST_ > 0.29 and D_est_ > 0.35. No loci had excess MD ancestry.

As with the candidate loci identified using genetic differentiation only, the loci identified using genomic cline analysis were in genes associated with immune function as well as general biological processes [72]. While we expected loci with excess ancestry to also exhibit steeper transitions from one parental population to the other, none of these loci had significantly steeper clines nor did any of the other 86 loci (Fig. 5b). Four loci did exhibit relatively shallower clines, indicative of balancing selection.

## Discussion

In this study, we genotyped SNPs in protein-coding genes to examine hybridization dynamics and selection pressures on a deer hybrid swarm. Genetic analyses on SNPs as well as mtDNA and microsatellites revealed the presence of two main population clusters, corresponding to BTD and MD. Mitochondrial divergence was high between the two lineages, far exceeding levels typically observed between sister species [52, 75]. Despite high mtDNA divergence, admixture analyses showed the presence of hybrid swarm with extensive bi-directional hybridization and hybrids extending beyond the F1 generation. Although we predicted that SNPs in genes associated with mate choice would be under greater selection and purposely included a high proportion of candidate genes, we only found evidence of selection at a handful of loci. This suggests that species boundaries, though porous, are maintained by many loci each having a small effect. The swarm showed signatures of long-term stability, as evidenced by coincidence of clines across marker types [5], and is predicted to persist into the future.

### Genetic structure

Despite extensive hybridization, we found strong evidence to support the distinction of BTD and MD as evolutionarily independent lineages. All genetic marker types supported classifying individuals into two groups (BTD and MD), one on either side of a boundary located just east of the Cascades mountain range. The Cascade mountain region also serves as a genetic boundary for other species [76, 77]. As with previous studies on *Odocoileus* [46, 50, 51], the mitochondrial divergence between lineages we observed was comparable to or larger than that typically reported between mammalian sister species [52, 75].

We also observed a high degree of genetic differentiation between BTD and MD lineages for microsatellites and SNPs. Overall, both datasets yielded consistent assignments, with differences exclusively between pure and hybrid categories and not between parental categories. Disparate assignments of individuals for the two datasets could arise if hybridization frequency varied over time, for example if hybridization occurred thousands of years ago but not recently of vice versa. The concordant assignments we observed, using rapidly evolving microsatellites and more slowly-evolving SNPs, suggest stability of genetic structure over time. In this study, SNPs were chosen from conserved exons, a consequence of using the genome of a distantly related species (*Bos taurus*) to develop baits for exon capture. Using highly conserved genes between such closely related lineages can lead to an underestimation of genetic divergence. However, within these exons, we chose SNPs that showed high variability between lineages [78], with a focus on SNPs predicted to be under selection. This was done to increase our ability to discriminate between BTD and MD and may have caused us to overestimate divergence. Though the magnitude of differentiation calculated for SNPs was likely affected by our methodology, the overall pattern of divergence is consistent with morphological differences and current taxonomy (and microsatellite differences). 

We expected to see a higher proportion of hybrids resulting from matings between bucks from the lineage with larger body size (MD) and does from the lineage with smaller body size (BTD). Mating success has been correlated with larger male body size in cervids [79–81], including in other populations of *Odocoileus* [82–84]. However, we found both crosses to be equally common based on microsatellites, while SNP analyses suggested that MD doe (larger lineage) and BTD buck (smaller lineage) crosses were slightly more frequent. One explanation for our findings is that while MD does may have a weaker preference for BTD bucks than BTD does for MD bucks, the former cross may have higher reproductive success than the latter. Asymmetric reproductive success has been observed in other hybrid systems [30, 85–87]. Since hybrids are presumably intermediate in size, small BTD does carrying hybrid offspring could experience extra physiological stress, causing increased mortality prior to parturition [88, 89].

Alternatively, observed hybridization rates may be driven by population demography. Previous work on hybridization in cervids has attributed higher realized rates of hybridization between does of the larger species and bucks of the smaller species to differences in migration rates and population densities [58, 90–92]. If long distance migration does bias hybridization rates in our system, it seems unlikely to have a strong effect. We observed few migrants (based on STRUCTURE and fastSTRUCTURE analyses) overall and there were a similar number of migrants per lineage. Detailed demographic data for hybridizing populations of BTD and MD, in conjunction with data on mating attempts and offspring viability, would provide the data necessary to distinguish among these hypotheses for realized symmetrical gene flow in this system.

### Signatures of SNP selection

Though signatures of selection can be difficult to disentangle from the effects of demographic processes, the consistently high effective population sizes of BTD and MD [46] make this system powerful for detecting genes under strong selection. We predicted that selection would be strongest for SNPs in genes coding for proteins related to olfaction and immune function, which are important for mate choice in BTD and MD [47, 49]. A disproportionate number of immune and sensory related genes have been observed to be under selection in other mammals, including sheep [93, 94], cattle [95], and wolves [96]. Because of this pattern coupled with our SNP sampling bias towards putative candidate genes, we also expected to find a higher proportion of outlier loci compared to studies analyzing genome-wide SNPs (5–10%; reviewed in [97]). However, outlier detection methods only identified one SNP likely under selection. This SNP is located in EIF4G3, a gene part of a protein complex primarily involved with recruiting ribosomes to mRNA [65, 66]. Mutations in EIF4G3 have been linked to male-limited fertility in mice [70]. If EIF4G3 does play a role in mammalian reproductive isolation, it could explain why it was detected as an outlier in our study as well as in an European rabbit hybrid zone [32]. Fertility data for bucks, particularly F1 hybrids, will be key for testing whether there is an association between sterility and EIF4G3 genotypes in *Odocoileus*.

Our candidate gene approach did not cover all parts of the genome and it is possible that strong selection (major gene effects) exists at coding or non-coding loci we did not sample [98]. However, selection does not necessarily need to be strong in order to have a significant impact on gene flow. Multiple loci under weak selection can each have a small effect on restricting gene flow, creating a large effect overall [34, 99]. This polygenic scenario is supported in our study, where cline analysis of individual SNPs indicated that selection on the majority of SNPs was weak despite high overall genetic divergence between lineages. Of the ten loci with the highest genetic differentiation between lineages or that were identified as potential candidate loci in the *bgc* analysis, eight exhibited non-synonymous mutations and four were in putative mate choice genes. Since mutations in ROPN1L affect sperm motility and can cause male infertility in mammals [100], it is possible that selection on this gene in *Odocoileus* could represent a post-mating reproductive barrier. Genome-wide association studies would help reveal how mutations in these genes affect deer survival. The gene SCRG1 is associated with prion infection response (e.g. chronic wasting disease [101]); however, the SNP examined in this study is a synonymous mutation and therefore is unlikely to have an effect on immune function.

Contrary to our predictions, signatures of selection were also observed for genes associated with general cell processes. One explanation for selection on general cell process genes is environmental adaptation. For example, the pattern of divergence at the metabolism gene PLIN2 mimics the sharp ecological transition along the contact zone and could reflect the significant differences in diet that exist between BTD and MD [47, 102]. Genotyping SNPs in genes adjacent to the candidate loci identified in this study would help determine whether the patterns of weak selection we observed for general cell process genes could be explained by linkage to un-examined genes that are under selection [28, 38, 41]. It is also important to note that all of our hypotheses were made under the assumption that the exons analyzed in our study were analogous to those in the *Bos taurus* reference genome [103]. It is possible that these genes have additional or alternate functions in *Odocoileus* that would be expected to be under selection. A complete cervid genome and a higher density of SNPs distributed across the genome, including non-coding regions, would allow us to better understand the processes driving selection and test whether genes involved in mate choice or environmental adaptation are overrepresented among loci under selection.

### Hybrid zone dynamics

While the presence of distinct lineages on either side of the hybrid zone indicates that partial barriers to gene flow do exist, it is unclear what specific barriers are driving hybrid zone dynamics. We predicted that reproductive barriers would be important for restricting introgression across the hybrid zone but did not find strong evidence to support this hypothesis. Selection on individual putative mate discrimination genes was weak or non-existent. Moreover, if selection on reproductive barriers were strong, then we would expect these highly mobile bucks to disperse to mate with a doe from the same lineage. Instead, we found evidence of extensive bi-directional introgression, suggesting that reproductive barriers have been eroded or never existed between these lineages.

Physical geography also does not appear to have a strong effect on gene flow. If the Cascades range functioned as a significant barrier, then genetic cline centers would have aligned with ridgeline. Instead, the observed cline centers correspond with the interface between two distinct habitats [104], suggesting that ecology may be shaping the structure of the hybrid swarm. BTD habitat in western Oregon primarily consists of wet, montane forest while MD habitat in eastern Oregon is dry, coniferous woodland [47, 102]. The transition from BTD to MD habitat is dramatic with minimum annual precipitation dropping from 1140 mm to 250 mm between ecoregions west and east of cline center [104]. Additional precipitation and temperature variables show concordant patterns across ecotones, with BTD habitats being consistently wetter with less fluctuation in temperature compared to MD habitats [105].

The Cascade Mountain region is a hybrid zone hot spot [106], and phylogenetic breaks have been identified in the same region in black-capped chickadees [77], hairy woodpeckers [76], and tree squirrels [107]. In the latter species, ecology is also thought to influence selection on phenotype [107, 108]. In situ ecological studies on habitat use would provide insight in the differences in habitat use between MD and BTD lineages. To determine the extent to which different aspects of ecology influence the structure of the BTD-MD hybrid zone, future work could compare selection pressures in areas with relatively steeper ecological gradients (i.e. central Washington) with those with shallower transitions (i.e. northern California).

The co-localization of genetic clines among markers suggests long-term stability of the BTD-MD hybrid swarm. Mitochondrial DNA, microsatellites, and exonic SNPs have different mutation rates and therefore provide insight into the genetic structure of the swarm at different time periods. If the position of the swarm had changed over time, we would have expected to see a shift in cline center between types of genetic markers. We did observe some variation in cline center among individual SNPs but the majority of SNPs showed a distinct change in allele frequency that coincides with the average cline center across SNPs. The sharp ecological transition across the hybrid zone may prove to be such a strong barrier that the swarm is trapped in a habitat suitability trough [109]. Habitat within and surrounding the hybrid zone is likely to have been stable since BTD and MD came into secondary contact *c.* 8000 BP [46]. No major uplifts have occurred in the Cascades range for 5 million years [110] and climatic conditions have not radically changed since the last glacial maximum [111]. Assuming environmental conditions remain stable, we predict that the center of the hybrid swarm will remain constant.

There is conflicting evidence in regards to whether the width of the hybrid swarm has changed over time. The significantly wider cline width for microsatellite markers compared to SNPs suggests that the swarm is expanding. Microsatellites reflect recent population structure; whereas SNPs in protein-coding genes tend to be more conserved and provide insight into more distant evolutionary history. Alternatively, swarm width may be stable and wider microsatellite clines might reflect poorer resolution or mutation model differences compared to SNPs [112, 113]. Being neutral markers, microsatellites are also predicted to spread more easily across the landscape than protein coding genes, which could give the illusion of hybrid swarm expansion. One method of reconciling these competing hypotheses would be to compare SNPs with different mutation rates to SNPs from non-coding genome regions. By removing the marker type as a confounding factor, direct comparisons across multiple time scales could be made to test for historic and contemporary fluctuations in swarm size and position.

## Conclusions

Stable hybrid swarms provide an excellent opportunity to investigate long term gene flow between genetically, and often morphologically, distinct lineages. In the case of the BTD-MD hybrid swarm, the boundary between these highly divergent lineages is porous, and is more closely aligned with the sharp ecological transition than the physical ridgeline of the Cascade range. Ecology can be a strong driver of hybrid swarm dynamics, and in this system ecologically-based selection is presumably acting on many genes, each with a small effect. Multivariate tests for polygenic selection on a set of high-density, genome-wide SNPs (Genome wide association study (GWAS) approach) could facilitate further testing of our polygenic selection hypothesis (e.g. [114]). A GWAS approach could also be used to gain additional insight into the phenotypic traits associated with differentiation between BTD and MD lineages. This work illustrates how genomic approaches can improve insights into mechanisms that maintain species boundaries in the face of widespread admixture.

## Methods

### Sampling and DNA extraction

Tissue samples were obtained from hunter-harvested *Odocoileus hemionus spp. (lymph*, *n* = 165; gum, *n* = 4; ear, *n* = 2; muscle, *n* = 1) along three latitudinal transects, spanning the state of Oregon in the United States (Fig. 1; Additional file 2: Figure S2; Additional file 3: Table S1). All animals were harvested as part of state regulated hunting seasons in 2000, 2003, and 2009–2011 and sampled by Oregon Department of Fish and Wildlife staff when heads were submitted to the state for disease testing. As all of our samples were obtained from hunter-harvested individuals, there was a bias toward bucks (84%). No animals were specifically killed for this study and all sampling followed the guidelines for the use of wild mammals in research from the American Society of Mammalogists [115]. Prior to analysis, samples were stored at − 80 °C in vials containing silica desiccating beads. Locality details were obtained from hunter reported GPS coordinates or location descriptions. Genomic DNA was extracted from tissue samples using a Qiagen DNeasy Blood and Tissue Extraction Kit (Qiagen, Hilden, Germany).

### Mitochondrial DNA sequencing and analysis

All samples were sequenced for a 583 bp portion of the mitochondrial control region. Following the protocol detailed in Latch et al. [46], we used the forward primer Odh-dloopF (5′ GAGCAACCAATCTCCCTGAG 3′) and either the reverse primer Odh-dloopR (5′ GTGTGAGCATGGGCTGATTA 3′) or Odh-dloopR2 (5′ GTGTGAGCATGGGCTGATTA 3′). When the latter reverse primer was used, we lowered the annealing temperature to 56 °C. PCR products were sequenced at the University of Wisconsin Biotechnology Center and Macrogen Corp. (Rockville, Maryland, USA) on an ABI3730xl DNA Analyzer. We aligned and manually edited sequences using GENEIOUS version 7.1.9 (Biomatters, Auckland, New Zealand, available at: http://www.geneious.com). Haplotypes matched those in GenBank previously observed in the Pacific Northwest (FJ189203-FJ189249, FJ189298-FJ189323) [46]. We re-amplified and re-sequenced 10% percent of samples (*n* = 17) to quantify sequencing error rates. We observed no differences in base calls between replicated sequences.

We analyzed mitochondrial DNA sequences using Bayesian and Maximum Likelihood methods of phylogenetic reconstruction in MrBayes version 3.2.5 and RAxML version 8.2.9 [116], respectively. Using MrModeltest version 2.3 [117], we assessed partitioning schemes and models of best-fit based on Akaike Information Criterion (AIC). Based on these results, we selected a GTR + I + Γ model for the Bayesian analysis and GTR+ Γ for the Maximum Likelihood analysis, per the suggestion of Stamatakis et al. [116]. For both methods of analysis, we included published sequences of *O. h. hemionus* (FJ188901 and FJ18911) and *O. virginianus* (JQ037851 and JQ037857) from outside the hybrid zone to more accurately designate mitochondrial clades as *O. h. hemionus* and *O. h. columbianus* and potentially identify individuals admixed with *O. virginianus*. Three published sequences of *O. h. columbianus* were already part of the original dataset. The following outgroups were selected from within Cervidae: *Alces alces* (JN632595); *Cervus elaphus* (NC007704); *Dama dama* (NC020700).

The Bayesian analysis was run for 6 million generations with a 20% burn-in. We performed two independent runs, each with four Markov chain Monte Carlo (MCMC) chains that were sampled every 500 generations. Average standard deviation of split frequencies (< 0.01) was used to confirm chain convergence. The Maximum Likelihood analysis was conducted with 100 bootstraps using RAxML-HPC2 on XSEDE [116] on the CIPRES Science Gateway [118]. We calculated uncorrected mean pairwise genetic distances between major lineages in MEGA 7.0.21 [119]. Within clades, we calculated nucleotide diversity (π) and haplotype diversity (H) using DnaSP version 5 [120].

### Microsatellite genotyping

We amplified nine microsatellite loci previously used by Latch et al. [45] to characterize hybridization in *O. hemionus* (Odh C, Odh E, Odh G, Odh K, and Odh O: [121], BM848: [122], C273 and T40: [123], RT24: [124]). We followed the PCR protocol described by Latch et al. [45] and amplified products were visualized at the University of Wisconsin Biotechnology Center on an ABI3700 DNA Analyzer. Genotyping was performed in GENEMARKER (SoftGenetics, LLC). We re-genotyped 10% of samples (*n* = 17) to quantify our genotyping error rate. We recorded a single instance of allelic dropout in 153 repeated genotypes, for a microsatellite genotyping error rate of 0.65%.

### Exon capture and SNP assay development

An initial exon capture was performed on three BTD and four MD showing no admixture based on microsatellite analysis [78]. Due to the lack of a complete cervid genome, the *Bos taurus* genome [72] was used to develop baits. These baits targeted exons across the cattle genome, a subset of which were candidate genes associated with immune function and reproduction. The exon capture was performed using a modified Agilent in-solution protocol to enrich for template DNA orthologous to the baits, which were then sequenced on a HiSeq sequencer (for details see [78]). Sequencing data were used to identify SNPs within exons and build consensus sequences for the regions flanking each SNP. These consensus sequences were used to develop end-point qPCR assays for SNP genotyping (for detailed methods for consensus sequence generation see [93]). For each SNP identified, expected heterozygosity was calculated across all samples and Weir and Cockerham’s F_ST_ [125] was calculated between MD and BTD using VCFtools [126]. Allele frequencies were also calculated at each locus for each species separately. Loci were removed if the mean phred scaled genotype likelihood or the mean genotype quality was less than 50.

We used multiple methods to select SNPs from the subset of seven individuals for downstream analysis. First, to enrich for loci that were informative for species delineation, we chose SNPs that were fixed in one (*n* = 60) or both subspecies (*n* = 2) and SNPs that had F_ST_ > 0.25 between lineages (*n* = 36). We conservatively chose F_ST_ > 0.25 in order to reliably identify hybrids [127]. SNPs were only retained if genotypes were called in all seven individuals, if the minor allele was observed more than three times across all individuals, if there were fewer than five Ns on the consensus sequence, and if no Ns were observed within 50 nucleotides of the SNP. Second, we selected SNPs that were likely to cause alterations to protein structure and function by selecting all transversions where F_ST_ > 0.25 (*n* = 5). We also chose transversions if all seven individuals were genotyped, if fewer than 10 Ns were observed on the consensus sequence, and if no Ns were observed within 50 nucleotides of the respective SNP (*n* = 35). Lastly, SNPs with expected heterozygosity between 0.2 and 0.6 that were deemed potentially informative for population assignment, population structure analysis, and estimation of relatedness were chosen for which all seven individuals were genotyped, if the minor allele was observed more than twice, if there were fewer than 5 Ns on the consensus sequence, and if no Ns were observed within 50 nucleotides of the SNP (*n* = 121).

A total of 154 SNPs and their corresponding consensus sequences passed at least one of set of filtering criteria. Of those loci, we submitted 130 for KASP-by-design Fluidigm assay design (LGC Genomics LLC, Beverly, Massachusetts), prioritizing loci showing evidence of being informative for species diagnostics and transversions. We tested an initial set of 95 assays by genotyping 47 samples from across the hybrid zone in duplicate. DNA concentration and quality was standardized in order to eliminate any effects associated with low sample quality or variable DNA concentration. Concentration and quality were assessed using a Nanodrop 2000 spectrophotometer (Thermo Fisher Scientific, Waltham, Massachusetts). For each sample, if the DNA concentration was less than 40 ng/μl or the 260/280 ratio was less than 1.8, then the DNA was precipitated using isopropanol, rinsed three times with 500 μl of 80% ethanol, then re-suspended in 15% of the original elution volume.

Samples were standardized to a concentration of 60 ng/μl prior to genotyping with Fluidigm 96.96 Dynamic Arrays and the Fluidigm EP1 Genotyping System (Fluidigm Corporation, San Francisco, California) using recommended reaction conditions for KASP KBD-Fluidigm Assays. Fluorescence intensity plots were examined for each assay using Fluidigm SNP Genotyping Analysis software. Genotypes were called using the no template control (NTC) normalization method to normalize the data against background noise and a 60% confidence threshold in the genotype assignment. Assays were retained if duplicate genotypes were concordant, genotype clusters were easily distinguishable, and assays yielded polymorphic genotypes. The remaining set of 35 assays was tested by genotyping 95 samples from across the hybrid zone in duplicate. If assays from the initial set of 95 were monomorphic or lacked homozygotes for the minor allele, they were re-tested using the same set of 95 samples used to test the second set of assays.

Of the 130 assays tested, 111 were variable and yielded high confidence genotypes with a high degree of concordance between duplicate PCRs. From the 111 assays that were successful, we included 95 SNPs for our final SNP genotyping panel that had well defined clusters (easy to score genotypes) and at least one occurrence of each possible genotype. We preferentially included assays targeting transversions, potential species diagnostic SNPs, and SNPs in genes of known function. This final set of assays was used to genotype all samples from across the hybrid zone using the same reaction conditions used for testing. To verify that assays were consistently yielding correct genotypes, we checked for concordance between exon capture and assay derived genotypes for four individuals that were genotyped using both technologies.

### Admixture analysis

We investigated the presence of hybridization by analyzing both the microsatellite and SNP datasets in the software programs STRUCTURE version 2.3.4 (microsatellites [128, 129]), fastSTRUCTURE version 1.0 (SNPs [130]) and NewHybrids version 1.1 (microsatellites and SNPs [131]). In both STRUCTURE and fastSTRUCTURE, a Bayesian algorithm was used to assign individuals to one or more clusters (K). The likelihood that a given individual belongs to a particular cluster is given by a Q value. Higher Q values indicate a greater posterior probability that an individual belongs to that cluster. All other individuals were considered hybrids [127]. In each program, we executed runs with a burn-in of 10^4^ iterations followed by 10^6^ iterations and performed ten replicate runs for K = 1 through K = 8. For the STRUCTURE analyses, we set the parameters to allow for admixture between clusters and selected the correlated allele frequency model. We assessed stationarity by ensuring that MCMC runs yielded a Gelman-Rubin statistic of less than 1.1 (calculated in R; [132]).

Using STRUCTURE HARVESTER [133], we combined runs for each value of K and estimated the most likely number of clusters based on the highest value of Δ K and where Ln(K) plateaued [128, 129]. In contrast, fastSTRUCTURE determines the most likely K in two ways. First, by calculating the value of K that maximizes marginal likelihood and then by calculating the minimum K needed to account for almost all of the samples’ ancestry. When the values of K selected by the two approaches are not equivalent, the user chooses the most biologically sound value of K. For each dataset, runs were combined for using CLUMPP [134] and visualised in Microsoft Excel. We iteratively re-ran the individuals assigned to each cluster in separate runs using the methods above to determine the presence of sub-structure.

We established thresholds for ‘pure’ parentals using simulated data. Since microsatellite and SNP data was not available for BTD and MD outside of the hybrid zone, we used individuals that met a stringent threshold of Q ≥ 0.95 for either the black-tailed deer or mule deer cluster as proxies for allopatric populations. These individuals were used to simulate 500 genotypes of ‘pure’ parentals in HYBRIDLAB [135]. The simulated genotypes were analysed in STRUCTURE (microsatellites) and fastSTRUCTURE (SNPs) for K = 2 using the same parameters as described above. We calculated the 95% confidence intervals of the distribution of Q values for parentals from the STRUCTURE and fastSTRUCTURE analyses and applied these intervals to the empirical data to classify individuals as pure BTD, pure MD, or hybrid.

For each of the three groups (BTD, MD, hybrid), we performed AMOVAs between pairs of transects using GENODIVE. The microsatellite and SNP data was analysed separately. We analyzed each locus separately and assessed significance at α = 0.05, following false discovery rate correction [136].

As with STRUCTURE and fastSTRUCTURE, NewHybrids uses a clustering algorithm to calculate the probability of an individual belonging to either parental group. Additionally, NewHybrids calculates the probability that an individual belongs to one of four hybrid classes (F1, F2, and backcrosses). Models for allele frequencies and mixing proportions were implemented with a Jeffrey’s-like prior and run ten times with 10^6^ sweeps and a burn-in of 10^4^ sweeps. This process was repeated using Uniform priors. All results were summarized in CLUMPP and visualized in Microsoft Excel. We ran the simulated individuals used in the STRUCTURE and fastSTRUCTURE analyses in NewHybrids using the same parameters. We then calculated the 95% confidence intervals of the distribution of probabilities that simulated ‘pure’ individuals belonged to one of the parental groups. These confidence intervals were used to establish cut-offs for parental group assignment for the empirical data. Remaining individuals were considered hybrids.

### Allelic diversity

For both the microsatellite and SNP datasets, we quantified allelic diversity for putative parental subspecies and hybrids based on the corresponding STRUCTURE and fastSTRUCTURE results (see below). This included the number of alleles and private alleles, expected and observed heterozygosity, and deviation from random mating (F_IS_). We calculated F_ST_ and Jost’s *D* [62] using corrected average H_S_ and H_T_ across loci as recommended by Meirmans and Hedrick [137]. Measures of allelic diversity and genetic differentiation were calculated in GenAlEx [138], with the exception of F_IS_ which was calculated in Genodive [139]. In Arlequin, we tested for linkage disequilibrium between microsatellite loci only and applied a false discovery rate correction to determine statistical significance [140].

### Outlier detection and cline analyses

We identified outlier loci using pcadapt [141] implemented in R (R Development Core [142]) and BayeScan 2.1 [143]. We only analyzed ‘pure’ BTD and ‘pure’ MD and screened for loci with minor allele frequencies < 0.05. Only one locus had a minor allele frequency < 0.05 (MAF = 0.008) and due to its near fixation in both parental groups, we chose to exclude this locus. The R package pcadapt uses a principal component analysis (PCA) to identify loci strongly associated with population structure and presumably under selection. We performed a PCA and chose the number of axes to retain in further analyses based on a scree plot, which shows the total variance in the data represented by each PC. We then calculated the Mahalanobis distance test statistic for each locus [144]. The *p* values associated with each test statistic were converted to q values to account for false discovery rates using the R package qvalue version 2.4.2 [145] and the threshold for statistical significance was set to α = 0.05. BayeScan estimates the posterior probability that a locus is presumably under selection and test for departures from neutral expectations by comparing allele frequencies within populations to the entire dataset. We ran BayeScan using the default parameters and applied a cut-off of Q < 0.05 to determine statistical significance.

We performed geographic cline analyses to investigate where the transition from genetically BTD to genetically MD occurred in relationship to the Cascades ridgeline, the assumed BTD-MD boundary. We fit geographic clines to each of the three genetic datasets as well as individual SNPs using a Metropolis-Hastings Markov chain Monte Carlo algorithm executed in the R package HZAR version 0.2–5 [146]. All individuals (parentals and hybrids) were included in the analyses. To generate clines, we first converted the two dimensional geographic locations of each sample to a one dimensional transect perpendicular to the Cascades ridgeline. We calculated the distance from each sample to the nearest point along the ridgeline using the ‘Near’ function in ArcGIS version 10.3.1. Mitochondrial haplotype data was coded as 1 or 0, with 1 indicating the haplotype was found within the BTD mitochondrial clade. For the mitochondrial dataset, we chose a model with maximum and minimum values fixed to 1 and 0, respectively, and did not estimate cline tails. Clines were fitted to the hybrid index (Q value) calculated in STRUCTURE or fastSTRUCTURE for the microsatellite and SNP data, respectively.

Cline analysis was also performed separately for each SNP using the observed genotypes. We selected models that allowed for the minimum and maximum values of the cline to be fixed based on the observed data (fixed) or allowed to vary (free) and tails were either not estimated (none) or both estimated independently (both). The three models tested (model 1: fixed/none; model 2: fixed/both; model 3: free/both) were compared to the null model using corrected Akaike information criterion (AICc). The model with the lowest AICc was considered the best model and used for further analysis. When the AICc for the null model was 2 or more units lower than the AICc for the best model, the null model was rejected. Using the selected models, we calculated cline center and width for all markers and determined coincidence using confidence intervals of two log-likelihood scores. Slope was calculated by dividing the change in actual or estimated allele frequency by cline width. Sharper slopes are associated with stronger selection whereas shallower slopes are indicative of weaker selection.

To complement the geographic cline analysis, we also performed genomic cline analyses specifically test for outlier loci. We used the software bgc [147, 148] to generate clines using genotypes for hybrid individuals because it does not require fixed alleles in parental populations. The cline parameter α reflects excess ancestry from one of the two parental populations. A shift in the cline center (α > 0 or α < 0) indicates that individuals have greater ancestry from one of the parental populations than expected. The β parameter indicates the rate of transition across the hybrid zone from low to high probability of belonging to one of the parental populations, corresponding to cline steepness. Loci under strong positive selection are expected to have high values of β and high absolute values of α, resulting in steep clines that are offset from average cline center. Loci not under selection are expected to have values of α and β that do not deviate from neutral expectations.

For the genomic cline analysis, the parental populations comprised individuals identified as putative parentals in the SNP fastSTRUCTURE analysis (BTD: Q > 0.865; MD: Q > 0.899). All other individuals were considered hybrids. Following the protocol of Trier et al. [74], we ran five independent runs with 100,000 MCMC and a burn-in of 25,000, retaining values from every fifth iteration. The maximum deviate from the uniform for proposed hybrid index (u) was changed to 0.001 and all other parameters were set to the default values. We calculated the Gelman-Rubin convergence diagnostic to assess stationarity in R [132]. Since all runs were quantitatively similar (all scale reduction factors < 1.04), we present the results from the run with highest log-likelihood and therefore the best fit for our data. SNPs were classified outliers if the 95% credibility interval of α and/or β excluded zero and there was > 0.5 difference in allele frequency between parental populations. Thus, SNPs with excess BTD or MD ancestry and/or that show a sharp change in allele frequency between parental populations were not considered outliers when the difference in allele frequencies between parental populations was small (< 0.5).

## Additional files

Additional file 1: **Figure S1.** Maximum likelihood tree based on mitochondrial DNA control region haplotypes. Bootstrap values > 0.70 are provided next to internal nodes. The bar chart indicates the assignment of each individual as a black-tailed deer (blue), hybrid (purple), or mule deer (red) based on data from microsatellites (column 1) and SNPs (column 2). Outgroups and mule deer samples collected outside the hybrid zone are designated by GenBank numbers. MD = *Odocoileus hemionus hemionus*; WTD = *Odocoileus virginianus*. (PDF 469 kb)

Additional file 2: **Figure S2.** Collection localities for all *Odocoileus* individuals. Individuals are classified as black-tailed deer (blue circles), hybrids (purple squares), or mule deer (red triangles) based on A) mitochondrial clades and B) microsatellite STRUCTURE analysis. The Cascades ridgeline is indicated by the bold black line. Map source: Esri. (PDF 326 kb)

Additional file 3: **Table S1.** Sample information for all samples used in this study. Data include the group to which each sample was assigned, using mtDNA, microsatelite, or SNP data. (PDF 55 kb)

## Data Availability

Datasets supporting the conclusions of this article are available in the Additional file associated with this publication and at Figshare (10.6084/m9.figshare.9273248).

## Abbreviations

AICc: Corrected Akaike information criterion
BTD: Black-tailed deer
F_IS_: Deviation from random mating
GWAS: Genome-wide association study
K: the number of populations
LGM: Last glacial maximum
MCMC: Markov chain Monte Carlo
MD: Mule deer
mtDNA: Mitochondrial DNA
Q: the proportion of an individual’s ancestry from a population
SNPs: Single nucleotide polymorphisms
