# Supplementary material for: Evidence for adaptive introgression of exons across a hybrid swarm in deer
Source: BMC Evol Biol. 2019 Nov 4;19:199. doi: 10.1186/s12862-019-1497-x (PMC6827202; doi:10.1186/s12862-019-1497-x)
Supplement: Supplementary file 1 — Figure S1. Maximum likelihood tree based on mitochondrial DNA control region haplotypes. Bootstrap values > 0.70 are provided next to internal nodes. The bar chart indicates the assignment of each individual as a black-tailed deer (blue), hybrid (purple), or mule deer (red) based on data from microsatellites (column 1) and SNPs (column 2). Outgroups and mule deer samples collected outside the hybrid zone are designated by GenBank numbers. MD = Odocoileus hemionus hemionus; WTD = Odocoileus virginianus. (PDF 469 kb) [file 12862_2019_1497_MOESM1_ESM.pdf]

Fig S1 Maximum likelihood tree based on mitochondrial DNA control region haplotypes. Bootstrap values > 0.70 are provided next to internal nodes. The bar chart indicates the assignment of each individual as a black-tailed deer (blue), hybrid (purple), or mule deer (red) based on data from microsatellites (column 1) and SNPs (column 2). Outgroups and mule deer samples collected outside the hybrid zone are designated by GenBank numbers. MD = *Odocoileus hemionus hemionus*; WTD = *Odocoileus virginianus*

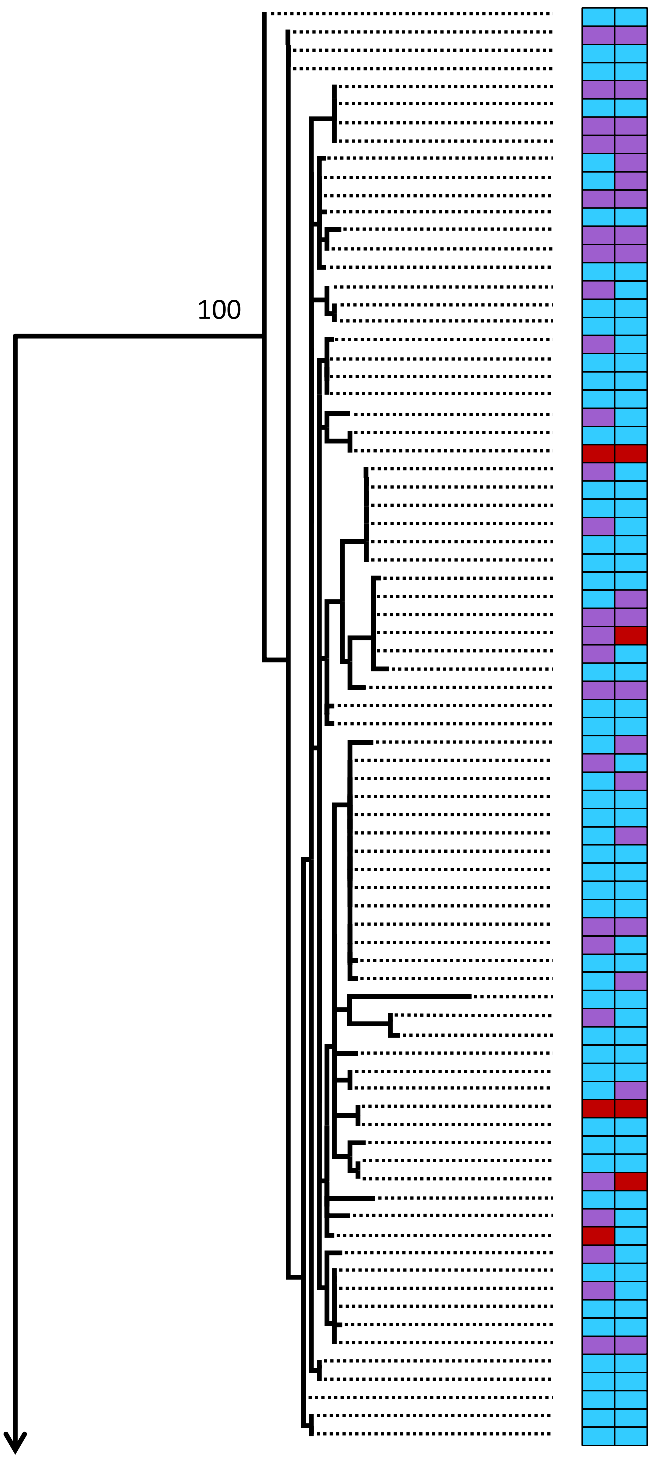

Continued on next page

Continued on previous page

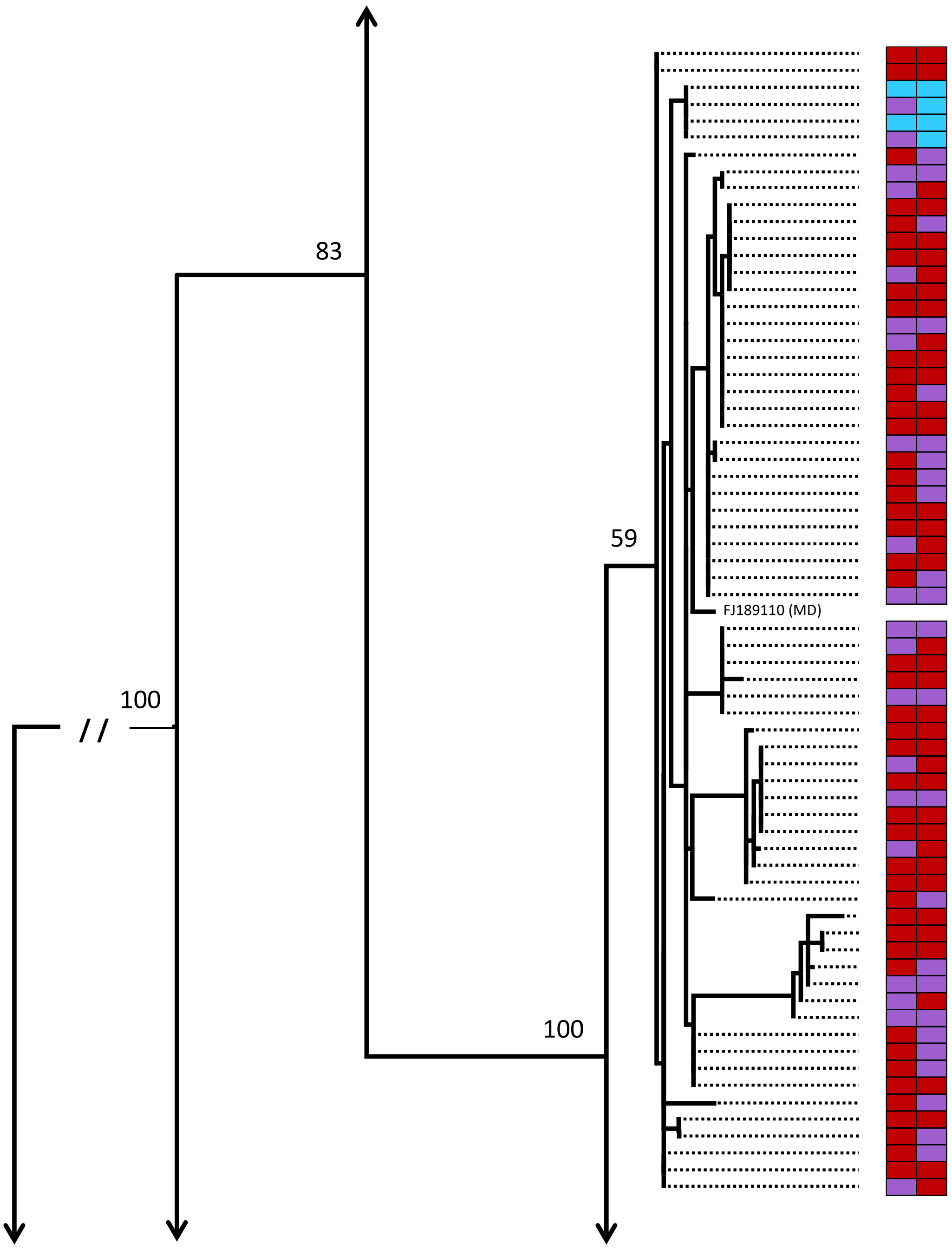

Continued on next page

Continued on next page

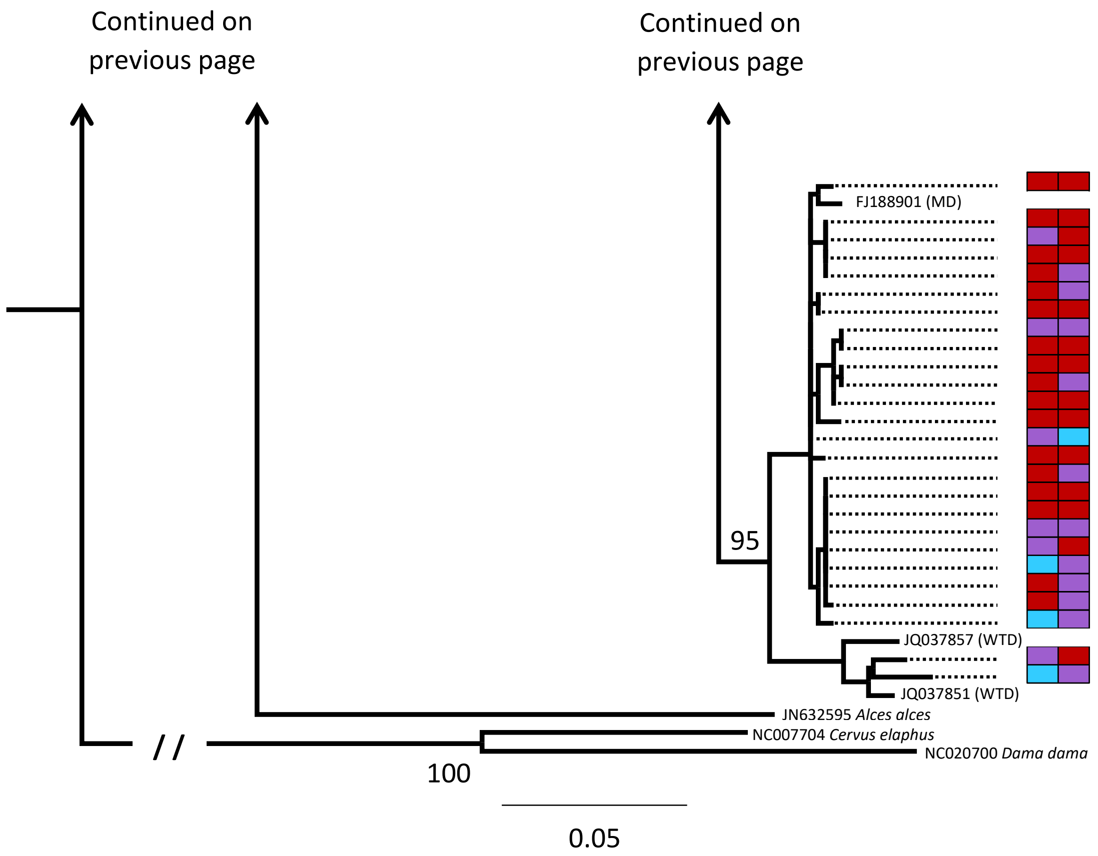
Continued on previous page
Continued on previous page
FJ188901 (MD)
95
JQ037857 (WTD)
JQ037851 (WTD)
JN632595 Alces alces
NC007704 Cervus elaphus
NC020700 Dama dama
100
0.05
