# Supplementary material for: Evidence for adaptive introgression of exons across a hybrid swarm in deer
Source: BMC Evol Biol. 2019 Nov 4;19:199. doi: 10.1186/s12862-019-1497-x (PMC6827202; doi:10.1186/s12862-019-1497-x)
Supplement: Supplementary file 2 — Figure S2. Collection localities for all Odocoileus individuals. Individuals are classified as black-tailed deer (blue circles), hybrids (purple squares), or mule deer (red triangles) based on A) mitochondrial clades and B) microsatellite STRUCTURE analysis. The Cascades ridgeline is indicated by the bold black line. Map source: Esri. (PDF 326 kb) [file 12862_2019_1497_MOESM2_ESM.pdf]

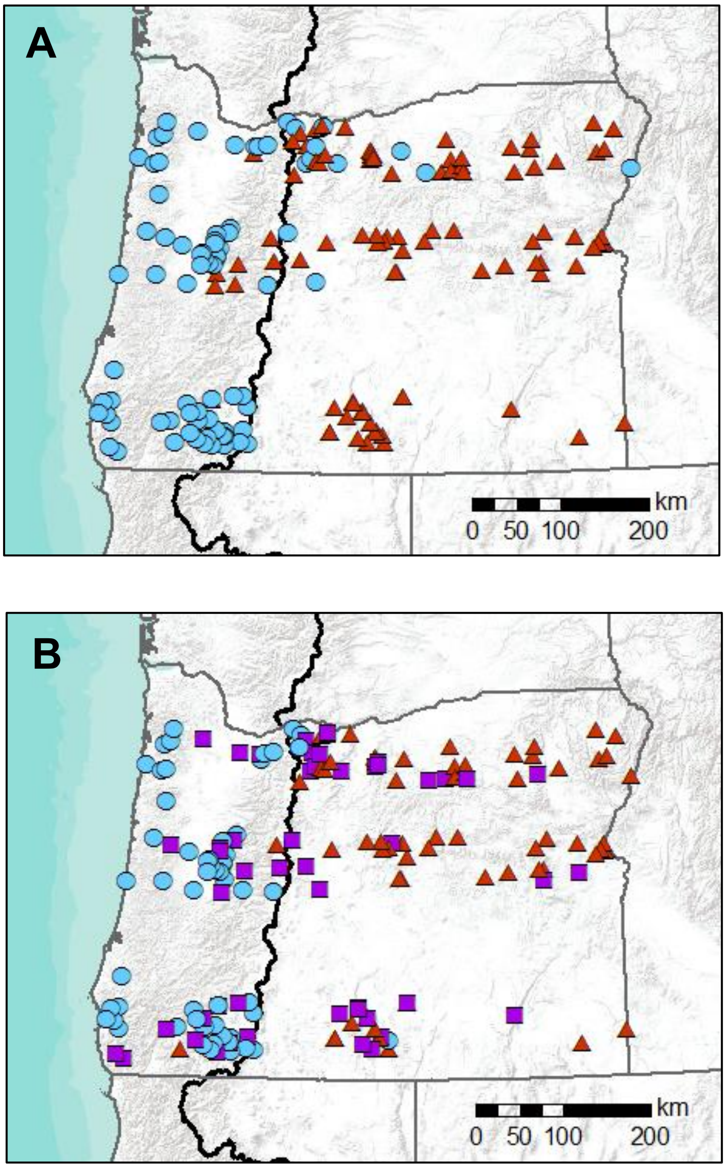


**Fig. S2** Collection localities for all *Odocoileus* individuals. Individuals are classified as black-tailed deer (blue circles), hybrids (purple squares), and mule deer (red triangles) based on A) mitochondrial clades and B) microsatellite STRUCTURE analysis. The Cascades ridgeline is indicated by the bold black line. Map source: Esri.
