# Supplementary material for: Evidence for adaptive introgression of exons across a hybrid swarm in deer
Source: BMC Evol Biol. 2019 Nov 4;19:199. doi: 10.1186/s12862-019-1497-x (PMC6827202; doi:10.1186/s12862-019-1497-x)
Supplement: Supplementary file 3 — Table S1. Sample information for all samples used in this study. Data include the group to which each sample was assigned, using mtDNA, microsatelite, or SNP data. (PDF 55 kb) [file 12862_2019_1497_MOESM3_ESM.pdf]

Table S1. Sample information for all samples used in this study. Data include the group to which each sample was assigned, using mtDNA, microsatelite, or SNP data.

Sample ID	Sex	Year	Latitude	Longitude	mtDNA	Microsatellites	SNPs
BTD01	Male	2000	44.833333	-123.611667	BTD	BTD	BTD
BTD02	Female	2000	44.452778	-123.766667	BTD	BTD	BTD
BTD04	Male	2003	43.008998	-124.146491	BTD	BTD	BTD
C001	Male	2011	44.345237	-117.199777	MD	MD	MD
C002	Male	2011	44.322356	-117.215451	MD	MD	MD
C003	Male	2011	44.400826	-117.245733	MD	MD	MD
C004	Male	2011	44.35	-117.3	MD	MD	MD
C005	Male	2011	44.304399	-117.369143	MD	MD	MD
C006	Male	2011	44.122085	-117.615067	MD	Hybrid	MD
C007	Male	2011	44.4225847	-117.6210389	MD	MD	MD
C008	Male	2011	44.481332	-118.072397	MD	MD	MD
C009	Male	2011	44.162143	-118.115881	MD	Hybrid	Hybrid
C010	Male	2011	44.04811	-118.119252	MD	Hybrid	MD
C011	Male	2011	44.1626149	-118.1790269	MD	MD	MD
C012	Male	2011	44.381835	-118.230576	MD	Hybrid	MD
C014	Male	2011	44.1336819	-118.6324181	MD	MD	MD
C015	Male	2011	44.0978259	-118.966712	MD	MD	MD
C016	Male	2011	44.51	-119.34	MD	MD	MD
C018	Male	2011	44.1848528	-119.6074132	MD	MD	MD
C019	Male	2011	44.513354	-119.655894	MD	MD	MD
C020	Male	2011	44.4113964	-119.765188	MD	MD	MD
C021	Male	2011	44.3138055	-120.091979	MD	MD	MD
C022	Male	2011	44.4598167	-120.1534273	MD	MD	MD
C023	Male	2011	44.093836	-120.167963	MD	MD	MD
C024	Male	2011	44.089816	-120.185213	MD	Hybrid	MD
C025	Male	2011	44.4593497	-120.2947139	MD	MD	MD
C026	Male	2011	44.4000254	-120.3562801	MD	MD	MD
C027	Male	2011	44.3837179	-120.4359094	MD	Hybrid	Hybrid
C029	Male	2011	44.3115012	-120.5960954	MD	MD	Hybrid
C030	Male	2010	44.4691825	-120.6564559	MD	Hybrid	MD
C031	Male	2011	44.3841571	-121.1582887	MD	Hybrid	Hybrid
C032	Male	2010	44.211	-121.533	MD	MD	Hybrid
C033	Male	2009	44.4252532	-121.9780788	MD	Hybrid	Hybrid
C034	Female	2010	44.1487744	-122.4158814	MD	BTD	BTD
C035	Female	2010	43.952	-122.455	BTD	BTD	BTD
C036	Male	2011	44.5101887	-122.5712796	BTD	Hybrid	BTD
C037	Female	2009	44.4660814	-122.5912496	BTD	BTD	BTD
C038	Male	2009	44.3087508	-122.7135831	BTD	BTD	BTD
C039	Male	2009	44.1638644	-122.7174947	MD	BTD	BTD
C040	Female	2009	44.0484451	-122.7349776	MD	Hybrid	BTD
C041	Male	2010	43.9170488	-122.7352272	MD	Hybrid	BTD
C042	Male	2009	44.181115	-122.748197	BTD	Hybrid	Hybrid

C043	Male	2011	44.2073243	-122.7542942	BTD	Hybrid	BTD
C044	Female	2010	44.2073243	-122.7542942	BTD	BTD	Hybrid
C045	Female	2009	44.2218352	-122.7545022	BTD	Hybrid	Hybrid
C046	Female	2011	44.4542042	-122.7731655	BTD	Hybrid	Hybrid
C047	Female	2011	44.3814113	-122.7942747	BTD	Hybrid	BTD
C048	Male	2011	44.3523773	-122.7943533	BTD	BTD	BTD
C049	Male	2010	44.1353411	-122.7944702	BTD	BTD	BTD
C051	Female	2009	44.2372353	-122.8749221	BTD	Hybrid	BTD
C052	Male	2009	44.1933951	-122.9357486	BTD	Hybrid	BTD
C053	Female	2011	44.2519222	-122.9551631	BTD	BTD	BTD
C054	Male	2009	44.2371737	-122.9761499	BTD	Hybrid	BTD
C055	Male	2011	44.120578	-122.9764227	BTD	Hybrid	BTD
C056	Male	2011	43.931584	-123.1576206	BTD	BTD	BTD
C057	Male	2011	44.32	-123.25	BTD	Hybrid	BTD
C058	Male	2011	44.3969578	-123.4862875	BTD	BTD	BTD
C059	Male	2011	44.017	-123.616	BTD	BTD	BTD
C060	Female	2009	43.9916933	-124.1309178	BTD	Hybrid	Hybrid
N001	Female	2011	45.2096341	-123.9150608	BTD	BTD	BTD
N002	Female	2011	45.1521823	-123.6981588	BTD	BTD	BTD
N003	Male	2011	45.4219865	-123.671476	BTD	Hybrid	BTD
N004	Male	2011	45.1664605	-123.6370777	BTD	BTD	BTD
N005	Female	2011	45.4550905	-123.5760799	BTD	BTD	BTD
N006	Male	2011	45.5838589	-123.5283273	BTD	BTD	BTD
N007	Male	2010	45.4984861	-123.0839348	BTD	Hybrid	BTD
N008	Male	2011	45.3670775	-122.5277452	BTD	Hybrid	BTD
N010	Female	2010	45.1793955	-122.3838136	MD	BTD	BTD
N011	Female	2011	45.2949488	-122.2399991	BTD	Hybrid	BTD
N012	Male	2011	45.367697	-122.2399423	BTD	BTD	BTD
N013	Male	2010	45.3674738	-122.1989946	BTD	Hybrid	BTD
N014	Male	2011	45.3820064	-122.0553522	BTD	BTD	Hybrid
N015	Male	2010	45.6291257	-121.7659599	BTD	BTD	BTD
N016	Male	2009	45.457189	-121.719137	MD	Hybrid	MD
N017	Male	2011	45.5560518	-121.6606414	BTD	Hybrid	Hybrid
N018	Male	2009	45.5121343	-121.5761058	MD	Hybrid	Hybrid
N019	Male	2009	45.189	-121.484	BTD	Hybrid	Hybrid
N020	Male	2009	45.3814173	-121.4714475	BTD	Hybrid	Hybrid
N021	Male	2011	45.251723	-121.4305691	MD	MD	Hybrid
N022	Male	2011	45.368632	-121.422651	MD	MD	Hybrid
N023	Male	2011	45.3672104	-121.3691991	MD	Hybrid	Hybrid
N024	Male	2011	45.561	-121.339	BTD	Hybrid	Hybrid
N025	Male	2011	45.2227585	-121.3281184	MD	Hybrid	Hybrid
N026	Female	2010	45.213	-121.281	BTD	Hybrid	MD
N027	Male	2011	45.581	-121.26	MD	MD	MD
N028	Male	2011	45.5987394	-121.2414283	MD	MD	Hybrid
N029	Male	2011	45.33	-121.13	MD	Hybrid	MD
N030	Male	2011	45.1928908	-121.0438885	MD	MD	MD

N031	Male	2011	45.580937	-120.905892	MD	MD	MD
N032	Male	2010	45.338757	-120.550735	MD	MD	Hybrid
N033	Male	2011	45.25645	-120.538161	MD	Hybrid	Hybrid
N034	Male	2010	45.297757	-120.531185	MD	Hybrid	Hybrid
N035	Male	2011	45.302292	-120.530405	MD	MD	MD
N036	Male	2010	45.324	-120.516	BTD	MD	MD
N037	Male	2011	45.300843	-120.510339	BTD	Hybrid	MD
N038	Male	2011	45.259	-120.507	MD	Hybrid	Hybrid
N039	Male	2011	45.1	-120.23	MD	MD	Hybrid
N040	Male	2011	45.33	-120.13	MD	MD	Hybrid
N041	Male	2011	45.096032	-119.770461	MD	MD	MD
N042	Male	2011	45.12	-119.51	MD	MD	MD
N044	Male	2011	45.13	-119.38	MD	Hybrid	MD
N045	Male	2011	45.18	-119.22	MD	MD	MD
N046	Male	2011	45.25	-119.22	MD	MD	MD
N047	Female	2011	45.25	-119.2	MD	MD	MD
N048	Male	2011	45.109318	-119.193812	MD	MD	MD
N049	Male	2011	45.357262	-118.493065	MD	Hybrid	MD
N050	Female	2011	45.0964246	-118.4506189	MD	MD	MD
N051	Male	2011	45.333575	-118.2312823	MD	MD	MD
N052	Male	2011	45.431916	-118.207766	MD	MD	Hybrid
N053	Male	2011	45.1527128	-118.1760736	MD	MD	MD
N054	Male	2011	45.4057185	-118.0865708	MD	MD	MD
N055	Male	2011	45.2023903	-117.8602757	BTD	MD	MD
N057	Male	2009	45.29	-117.27	MD	Hybrid	Hybrid
N058	Male	2009	45.309173	-117.158405	MD	MD	MD
N059	Male	2009	45.52	-117.01	MD	MD	Hybrid
N060	Male	2010	45.088957	-116.797112	MD	MD	MD
S001	Male	2011	42.48	-117.03	MD	MD	MD
S002	Male	2011	42.3456625	-117.6608627	MD	MD	Hybrid
S003	Male	2011	42.6604137	-118.5901395	MD	Hybrid	MD
S005	Male	2011	42.796186	-120.086613	MD	MD	Hybrid
S006	Male	2011	42.799239	-120.090475	MD	Hybrid	Hybrid
S007	Male	2010	42.33	-120.35	MD	MD	Hybrid
S008	Female	2011	42.4046723	-120.3851298	MD	BTD	Hybrid
S009	Male	2011	42.4479993	-120.4641143	MD	Hybrid	MD
S010	Male	2010	42.432	-120.475	MD	MD	Hybrid
S011	Male	2010	42.43	-120.48	MD	MD	Hybrid
S012	Male	2010	42.53	-120.55	MD	MD	MD
S013	Male	2010	42.3307799	-120.5803961	MD	Hybrid	Hybrid
S014	Female	2011	42.6642242	-120.6535322	MD	MD	MD
S015	Male	2011	42.6355564	-120.6583239	MD	Hybrid	MD
S016	Male	2011	42.368	-120.709	MD	Hybrid	Hybrid
S017	Male	2010	42.756	-120.785	MD	Hybrid	Hybrid
S018	Male	2011	42.7364421	-120.7907959	MD	Hybrid	MD
S019	Male	2010	42.591636	-120.866765	MD	MD	Hybrid

S020	Male	2010	42.6954106	-121.045387	MD	Hybrid	Hybrid
S021	Male	2010	42.43	-121.1	MD	MD	Hybrid
S022	Male	2010	42.4344106	-121.1052171	MD	MD	Hybrid
S023	Male	2010	42.431	-121.112	MD	MD	Hybrid
S025	Male	2011	42.7846567	-121.8287552	BTD	BTD	Hybrid
S026	Male	2009	42.2935531	-122.2564967	BTD	BTD	BTD
S027	Male	2010	42.6708701	-122.2759567	BTD	Hybrid	BTD
S028	Male	2009	42.4398657	-122.3170519	BTD	BTD	BTD
S029	Male	2011	42.7799916	-122.3419649	BTD	Hybrid	BTD
S030	Male	2009	42.3094362	-122.3561913	BTD	Hybrid	BTD
S031	Male	2009	42.7797523	-122.4604923	BTD	Hybrid	Hybrid
S032	Male	2010	42.2806022	-122.5712403	BTD	Hybrid	BTD
S033	Male	2009	42.4109875	-122.5907409	BTD	Hybrid	BTD
S034	Male	2009	42.3671211	-122.5910011	BTD	Hybrid	BTD
S035	Male	2010	42.3671211	-122.5910011	BTD	BTD	BTD
S036	Male	2009	42.3670799	-122.610809	BTD	BTD	Hybrid
S037	Male	2009	42.4327851	-122.69875	MD	Hybrid	BTD
S038	Male	2009	42.7436262	-122.7458299	BTD	Hybrid	BTD
S039	Male	2010	42.2653905	-122.7476277	BTD	BTD	BTD
S040	Female	2009	42.7435086	-122.7658853	BTD	Hybrid	BTD
S041	Male	2009	42.7435086	-122.7658853	BTD	BTD	BTD
S043	Female	2011	42.3455203	-122.8165369	BTD	BTD	BTD
S044	Male	2009	42.6072183	-122.8167316	BTD	BTD	BTD
S045	Male	2011	42.2801753	-122.8654195	BTD	Hybrid	BTD
S046	Female	2011	42.5128395	-122.9449282	BTD	BTD	Hybrid
S047	Female	2011	42.6147281	-122.9452911	BTD	Hybrid	BTD
S048	Female	2010	42.323523	-122.9637863	BTD	BTD	BTD
S049	Male	2011	42.3969654	-123.0622509	BTD	Hybrid	BTD
S050	Female	2010	42.6876978	-123.1025232	BTD	BTD	BTD
S051	Male	2010	42.2952196	-123.2592313	BTD	Hybrid	BTD
S052	Male	2011	42.5131228	-123.3374872	BTD	Hybrid	BTD
S053	Male	2011	42.4986862	-123.4747499	BTD	Hybrid	BTD
S054	Male	2011	42.176312	-124.0445208	BTD	Hybrid	BTD
S055	Male	2011	42.4754875	-124.1575785	BTD	BTD	BTD
S056	Male	2011	42.215	-124.161	BTD	BTD	BTD
S057	Male	2011	42.688	-124.161	BTD	BTD	BTD
S058	Male	2011	42.547452	-124.221133	BTD	BTD	BTD
S060	Male	2010	42.563022	-124.333716	BTD	BTD	BTD
